# Microglia across evolution: from conserved origins to functional divergence

**DOI:** 10.1038/s41423-025-01368-6

**Published:** 2025-11-21

**Authors:** Takashi Shimizu, Marco Prinz

**Affiliations:** 1 Institute of Neuropathology, Faculty of Medicine, University of Freiburg, Freiburg, Germany; 2 Signaling Research Centers BIOSS and CIBSS, University of Freiburg, Freiburg, Germany; 3 Center for Brain Research and Advancements In Neuroimmunology (BRAIN), Faculty of Medicine, University of Freiburg, Freiburg, Germany

**Keywords:** Microglia, species, ontogeny, evolution, Innate immunity, Neuroimmunology

## Abstract

Microglia, the resident immune cells of the central nervous system, exhibit conserved developmental origins and core molecular signatures across vertebrate species, highlighting their crucial importance in the central nervous system. While homeostatic microglia maintain similar functions during phylogeny—such as immune surveillance, debris clearance, and synaptic pruning—their morphology, gene expression, and responses to stimuli remarkably vary by species. These differences reflect evolutionary divergence shaped by factors such as lifespan, regenerative potential, and immune architecture. This review integrates current findings from basic vertebrates such as zebrafish, rodents, and nonhuman primates with those from humans to highlight conserved and divergent aspects of microglial biology throughout evolution. Integrating these evolutionary differences is crucial for translating mechanistic insights across model organisms and advancing microglia-targeted therapies for neurological disorders.

## Introduction

**Fig. 1:**
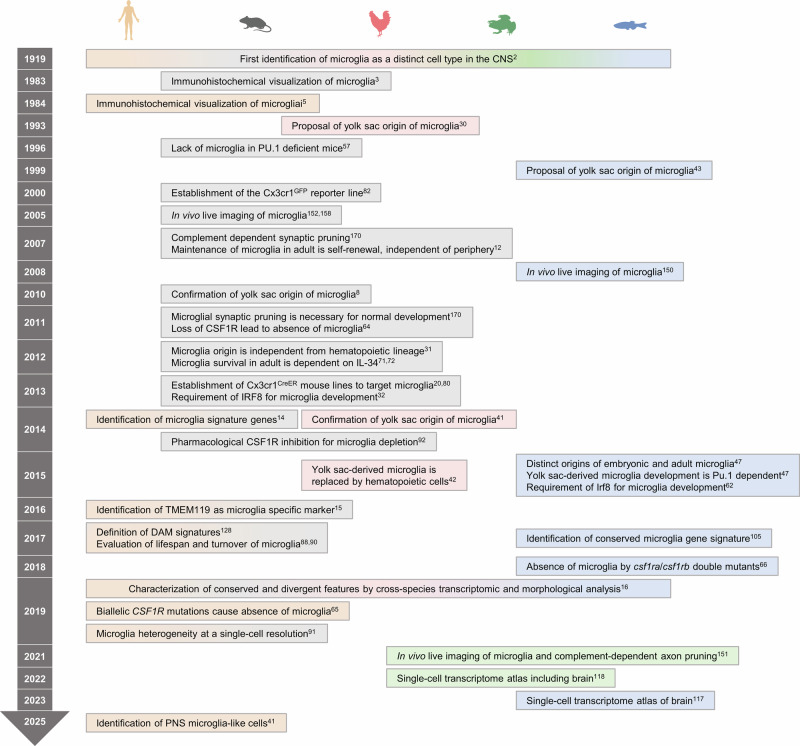
Timeline of key microglial research across species. This timeline summarizes major discoveries in microglial biology across five species—human, mouse, chicken, frog, and zebrafish. Background shading denotes the species associated with each finding. The timeline illustrates the progression of methodological advances and conceptual insights that have shaped the field over the past century

Microglia are the resident immune cells of the central nervous system (CNS), where they play critical roles in maintaining brain homeostasis, supporting neurodevelopment, and responding to injury and disease [1]. Early histological studies in the early 20th century identified microglia as cells with small cell bodies and highly branched processes; since then, they have emerged as central players in neuroimmune interactions [2]. The introduction of immunohistochemical techniques further enabled the precise visualization and classification of microglia in the vertebrate brain [3–5]. More recently, advances in high-resolution imaging, transcriptomic profiling, and genetic fate-mapping models have substantially refined our understanding of microglial identity, origin, and function [6, 7] (Fig. 1).

Microglia are now recognized as a distinct CNS-resident myeloid lineage, defined by several key developmental, molecular, and functional features. First, they originate from primitive yolk sac-derived macrophages that colonize the CNS early in embryogenesis, prior to blood–brain barrier formation [8–11]. Second, they persist throughout life within the CNS and maintain their population through self-renewal, independent of bone hematopoietic stem cell-derived monocytes [12, 13]. Third, they exhibit a conserved transcriptional signature characterized by the expression of genes such as *Tmem119*, *P2ry12*, *Sall1*, *Cx3cr1*, *Hexb*, and *Fcrls*, along with lineage-determining transcription factors such as *Spi1* and *Irf8* [14–16]. Fourth, microglia display a dynamic morphology, ranging from highly ramified surveillant forms to ameboid activated shapes [17, 18]. Fifth, they carry out specialized functions such as synaptic pruning, immune surveillance, phagocytosis, and modulation of neuronal circuits [19, 20].

On the basis of these combined features, microglia are believed to be conserved across all major vertebrate groups, including mammals, birds, reptiles, amphibians, and fish [21, 22]. While specific molecular markers and ontogeny may exhibit lineage-specific variations, these organisms share the core hallmarks of yolk sac–derived, self-renewing, CNS-resident macrophages with specialized gene expression profiles and critical developmental functions [16, 23].

In contrast, most invertebrates lack cells that exhibit the full spectrum of defining characteristics of vertebrate microglia. Some invertebrate species possess microglia-like cells—resident phagocytic or glial-like cells that contribute to CNS surveillance and debris clearance—but these cells typically arise from distinct developmental origins and lack the conserved molecular signature observed in vertebrate microglia [23, 24]. For example, in *Drosophila melanogaster*, embryonic hemocytes infiltrate the CNS and carry out phagocytic functions reminiscent of microglial activity; however, these cells originate from mesodermal lineages unrelated to yolk sac progenitors and do not express homologs of canonical microglial markers [25, 26]. Similarly, in *Caenorhabditis elegans*, certain glial-like cells support neural function, but there is no clear evidence that resident macrophage-like cells play equivalent immunological or developmental roles [27].

Thus, although CNS-associated phagocytes or glial cells are present in invertebrates, their distinct developmental origins, lack of long-term residency, and absence of conserved transcriptional programs distinguish them from bona fide microglia. These observations support the view that microglia represent a vertebrate-specific evolutionary innovation that potentially coemerged with the increasing complexity of the nervous system. In the following sections, we review the details of the individual components that define microglial identities across vertebrate species.

## Ontogeny

### Embryonic origin of microglia

Microglia arise early in development from progenitors located in the embryonic yolk sac [28, 29]. The yolk sac origin of microglia was first proposed in mice and chickens [9, 30] and was later confirmed through genetic lineage-tracing studies. Fate-mapping experiments elegantly demonstrated that these yolk sac–derived cells give rise to the entire microglial population in the brain without contributing from later bone marrow–derived monocytes [8]. Specifically, labeling embryonic progenitors at yolk sac stages—approximately embryonic day 7.5 (E7.5) in mice—results in labeled microglia in the adult brain, whereas labeling at later developmental stages does not. Moreover, mice lacking hematopoietic stem cells (HSCs) due to *Myb* mutation still develop a normal microglial population, further confirming their independence from definitive hematopoiesis [31]. Critically, c-kit^+^ erythromyeloid progenitors (EMPs) in the yolk sac serve as microglial precursors, whereas most other tissue macrophages arise from c-Myb^+^ definitive progenitors [32, 33]. These studies firmly established that primitive yolk sac-derived EMPs are the embryonic source of microglia in mammals, corresponding to the first wave of embryonic hematopoiesis [34].

Importantly, the yolk sac origin of microglia appears to be a conserved feature across vertebrates, albeit with species-specific variations. In humans, microglia are likewise thought to be derived from early yolk sac macrophages [21]. Immunohistochemical studies have detected the earliest microglia in the human forebrain at approximately 4–5 weeks of gestation, with these early cells dispersing throughout the developing cortex by the end of the first month [35, 36]. Time-resolved single-cell transcriptional and epigenetic profiling analyses of macrophages during prenatal development further support a yolk sac origin for human microglia [37–40].

In avian species such as chickens, a comparable embryonic wave of yolk sac–derived macrophages gives rise to primitive microglia in the developing brain. An early study reported the earliest appearance of macrophages in chicken embryos [30]. A transgenic chicken engineered with a macrophage-specific gene reporter confirmed that avian microglia originate from the yolk sac [41]. However, in contrast to those in mammals, these yolk sac-derived microglia in birds are transient; during later embryogenesis and after hatching, they are largely supplanted by incoming definitive hematopoietic cells [42].

In amniotic vertebrates, which lack a true extraembryonic yolk sac, microglial precursors emerge from analogous early hematopoietic sites. For example, in zebrafish embryos, primitive macrophages arise from the rostral blood island (RBI), which is analogous to the mammalian yolk sac [43]. These early macrophages differentiate near the yolk mass and migrate into the embryo proper, where they seed multiple tissues, including the brain [44, 45]. However, these embryonic microglia are eventually replaced by HSC-derived cells at the end of the juvenile stage [46, 47].

Thus, across vertebrate lineages—mammals, birds, and fish—the ontogeny of microglia begins with an embryonic source of myeloid progenitors, typically yolk sac-derived or its functional analog, that seeds the developing brain parenchyma. This ancestral strategy reflects the evolutionary conservation of microglia as a prenatally established, hematopoietic stem cell–independent population.

### Migration and colonization of the embryonic CNS

Following their emergence in the yolk sac or analogous region, microglial progenitors migrate into the developing CNS, where they colonize the embryonic brain. In mammals, this colonization begins remarkably early. In mice, yolk sac–derived precursors enter the neural tube around E9–E10 [8] shortly after the establishment of embryonic circulation [48]. Notably, there is strong evidence that microglial seeding in the brain occurs via vascular routes. In Ncx1-deficient mice, which lack a functional heartbeat and blood circulation [49], CNS macrophage invasion is absent despite normal yolk sac hematopoiesis, highlighting the dependence of early colonization on circulation [8]. However, extravascular routes are also essential for microglial development [50].

During human development, microglial precursors similarly infiltrate the brain during early organogenesis. Microglia have been identified in the embryonic human forebrain as early as 4.5 gestational weeks and initially cluster around entry sites such as the meninges, choroid plexus, and ventricular zones [35, 36]. From these sites, human microglia migrate both tangentially across the cortical surface and radially into deeper parenchymal regions. By 8–10 gestational weeks, microglia are broadly distributed throughout the fetal human brain [51]. Recent spatiotemporal transcriptomics studies showing the dynamics of human prenatal macrophages confirmed the presence of microglia in the brain at approximately 9 gestational weeks [39, 40].

Microglial colonization is particularly well visualized in lower vertebrates. In zebrafish, time-lapse imaging has captured primitive macrophages actively migrating into the developing brain [44]. These cells originate near the yolk sac, first enter the head mesenchyme, and then differentiate into early microglia in the brain and retina by approximately 60 hours post-fertilization [44]. Interestingly, this colonization can occur both in the presence [43] or absence of blood circulation [44], indicating that this migration is an active, circulation-independent developmental process.

A similar phenomenon has been observed in avian embryos. During chick development, yolk sac–derived macrophages invade the brain before full maturation of the cerebral vasculature occurs [30]. This independence from vascularization was further supported by embryonic parabiosis experiments [52]. These findings suggest that, in birds, microglial colonization of the CNS begins before the establishment of a fully mature cerebral vasculature.

Collectively, these studies underscore that microglial colonization of the embryonic CNS is a conserved, active, targeted migratory process. Across vertebrate species, progenitors enter the brain via permissive anatomical routes—such as the meninges, perivascular spaces, and ventricular zones—before dispersing broadly throughout the parenchyma.

### Core transcriptional regulators

The establishment and maintenance of microglial identity across vertebrate species are orchestrated by a combination of lineage-intrinsic transcriptional regulators and local CNS-derived cues [53]. Among the intrinsic regulators, the transcription factor PU.1 is indispensable for microglial specification and differentiation, representing evolutionarily conserved nodes of myeloid lineage commitment [29]. PU.1, encoded by *Spi1*, is a founding member of the E26-transformation-specific (ETS) family and acts as a master regulator of myelopoiesis [54, 55]. PU.1 binds to enhancer regions of myeloid genes and promotes the expression of macrophage-specific surface receptors and differentiation programs [56]. In PU.1-deficient mice, microglia and other macrophage lineages fail to develop despite the presence of primitive hematopoietic progenitors [57, 58]. This requirement is conserved in zebrafish, where *spi1b* knockdown abrogates early macrophage and microglial development [47]. Interferon regulatory factor 8 (IRF8) plays an equally pivotal role. It operates downstream of PU.1 and is required for the specification and maintenance of the microglial lineage [59]. In mice, IRF8 deficiency results in the downregulation of microglial identity genes [60] and disrupts homeostatic microglial morphology [61], highlighting its critical role in CNS macrophage specification.

This requirement is evolutionarily conserved in zebrafish, where Irf8 deficiency results in a complete absence of microglia and early macrophages, despite the presence of myeloid progenitors [62]. At later developmental stages, some macrophage populations partially recover, likely due to the onset of definitive hematopoiesis; however, microglia do not reappear [62], underscoring their specific dependence on early IRF8-mediated programs.

Together, PU.1 and IRF8 represent a conserved transcriptional axis governing the emergence of microglia from primitive progenitors. These intrinsic factors act in concert with CNS-derived signals to initiate and stabilize the microglial gene expression profile, enabling the cells to adopt their tissue-resident macrophage phenotype across diverse vertebrate species, including mammals and teleost fish.

### Environmental factors guiding differentiation

The differentiation of microglia is critically dependent on extrinsic signals from the CNS microenvironment. Among these, colony-stimulating factor 1 receptor (CSF1R) signaling is indispensable across species [63]. In mice, genetic ablation of Csf1r leads to a nearly complete absence of microglia [64], confirming its essential role in CNS macrophage ontogeny. This requirement is evolutionarily conserved. In humans, individuals with biallelic loss-of-function mutations in *CSF1R* show a complete absence of microglia in postmortem brain tissue, as documented in cases of pediatric-onset leukoencephalopathy [65]. A parallel phenotype has been observed in zebrafish double mutants lacking both Csf1ra and Csf1rb, which similarly fail to develop microglia [66], underscoring the conserved and nonredundant requirement for CSF1R signaling.

In contrast, CSF1–deficient mice, including Csf1^op/op^ mutants with a natural null allele, retain relatively normal numbers of microglia [67, 68]. This discrepancy suggests the involvement of an alternative ligand. Indeed, interleukin-34 (IL-34) was later identified as a second high-affinity ligand for CSF1R [69, 70]. IL-34 is expressed predominantly by neurons and is particularly important for maintaining microglia in the cortex and hippocampus, where its deletion causes regional microglial loss [71, 72]. This IL-34-mediated support is evolutionarily conserved, with comparable functions observed in the zebrafish CNS [73, 74] and avians in vitro [75].

Another essential environmental regulator is transforming growth factor–β (TGF-β), a cytokine enriched in the CNS that governs the transcriptional identity of microglia [76]. TGF-β signaling induces the expression of core microglial signature genes, and mice lacking TGF-β1 exhibit a reduced number of microglia or major disruptions in their homeostatic gene expression [14]. Similarly, treatment of human microglia with TGF-β1 induces homeostatic expression of microglial genes in vitro [77].

Together, these findings demonstrate that continuous signaling via the CSF1R and TGF-β pathways is fundamental for microglial development and survival. Whereas evidence for TGF-β signaling is currently limited to mammals, CSF1R signaling is conserved across vertebrates—including mammals, birds, and teleost fish—highlighting the deep evolutionary roots of microglial dependence on tissue-derived cues. Disruption of these environmental signals leads to severe depletion or dysfunction of microglia, underscoring their essential role as niche-derived instructors of microglial fate.

## Maintenance

### Self-renewal and autonomy of adult microglia

Microglia possess a remarkable capacity for long-term self-renewal in adulthood. Under physiological conditions, microglia across vertebrate species are maintained primarily through local proliferation, with minimal or no replenishment from peripheral monocytes [7, 28].

In mice, parabiosis studies first revealed that adult microglia self-maintain autonomously, with little to no recruitment from circulating myeloid precursors even after prolonged circulatory coupling [12, 13]. This autonomy is further supported by bone marrow chimera models using the CD45.1/CD45.2 markers, which confirmed that adult microglia are independent of hematopoietic cells under steady-state conditions [8].

This self-renewing autonomy is conserved beyond mammals. In birds, although embryonic microglia initially derive from yolk sac progenitors and are later replaced by definitive hematopoietic cells during late development, adult microglia become locally maintained thereafter, independent of further peripheral input [42]. Similarly, in zebrafish, microglia originate from both primitive and definitive hematopoietic sources, but once established, the adult population undergoes proliferation to replenish and maintain its pool [46, 47, 78].

Despite their differing embryonic origins, adult microglia from mammals, birds, and zebrafish share a core feature: their maintenance is governed by intrinsic self-renewal through stochastic local proliferation, with minimal reliance on peripheral hematopoietic input under steady-state conditions. This fundamental autonomy underscores a deeply conserved biological strategy for sustaining CNS immune surveillance.

### Microglial turnover

Microglial populations undergo continuous turnover, balancing local proliferation and apoptosis to maintain stable cells [79]. Recent studies have demonstrated that adult microglia are dynamic and exhibit species-specific turnover rates shaped by lifespan, brain region, and environmental cues.

In mice, fate mapping via CX3CR1-driven reporters revealed that microglia are long-lived cells, with labeled populations traceable for several months [80–84]. Similar data on microglial longevity were obtained with more microglia-specific mice, such as *Hexb*^CreERT2^ mice [85, 86]. Long-term in vivo imaging corroborates this finding, showing that individual microglia can persist for more than 15 months, with the population as a whole undergoing gradual remodeling although stochastic cell division and death [87]. BrdU incorporation assays estimated that approximately 0.5–2% of microglia proliferate at any given time under steady-state conditions, depending on the brain region, allowing the entire population to be renewed multiple times over the lifespan [88]. Notably, confetti-based clonal tracking revealed that microglial turnover occurs in a random manner during homeostasis [89]. This dynamic equilibrium ensures robust microglial maintenance and adaptability throughout life.

In humans, a similar proliferative fraction of approximately 2% Ki67^+^ microglia has been detected in both young and adult brain tissues [88]. However, owing to an extended lifespan, turnover proceeds at a significantly slower pace. Carbon-14 retrospective birth dating further revealed that human cortical microglia renew at an average annual rate of approximately 28%, translating to a median cellular lifespan of approximately four years [90]. Notably, a subset of human microglia has remained unchanged for decades; however, no permanent “immortal” subpopulation has been identified, as the majority of cells eventually undergo turnover [90]. This gradual renewal contributes to a heterogeneous age profile among human microglia [91], which may influence their functional properties over time.

In contrast, shorter-lived vertebrates exhibit faster microglial turnover. In zebrafish, the adult microglial pool is renewed multiple times throughout life, which is consistent with rapid tissue turnover in teleost species [74]. As in mammals, EdU and BrdU dual-pulse labeling has demonstrated that zebrafish microglia proliferate randomly during homeostatic conditions [78, 89].

Together, these findings illustrate that microglial maintenance strategies are closely associated with species-specific life history traits, with shorter-lived animals exhibiting more rapid turnover. Despite these differences in kinetics, the capacity for regulated renewal is a conserved feature of vertebrate microglia.

### Regulation of microglial maintenance

Transcription factors are essential not only for the development of microglia but also for their maintenance in adulthood. Recent studies have demonstrated a continued requirement for PU.1 in adult microglial survival. In adult mice, inducible deletion of PU.1 via the *Spi1*^fl/fl^; *Cx3cr1*^CreER^ system resulted in a marked reduction in the number of microglia within one week following tamoxifen administration [78]. In contrast, adult zebrafish with tamoxifen-induced Pu.1 deficiency alone presented no observable loss of microglia. However, when Spi-b, a paralog of PU.1, is deficient in zebrafish, microglia are rapidly depleted, indicating that Pu.1 and Spi-b play redundant roles in maintaining the adult microglial population [78]. These findings underscore a conserved molecular mechanism regulating microglial maintenance while highlighting species-specific differences in genomic architecture and transcription factor dependency.

Adult microglial survival is also critically dependent on extrinsic signals, particularly those derived from the CNS microenvironment. CSF1R signaling is indispensable in this context. Pharmacological inhibition of CSF1R in adult mice leads to rapid and nearly complete depletion of microglia within weeks, emphasizing its essential role in sustaining the adult population [92]. Similarly, CSF1R blockade in adult zebrafish and the postembryonic stage of frogs induces a profound reduction in microglia [93, 94], reinforcing the conserved necessity of CSF1R signaling for microglial homeostasis across species.

The reliance on CNS-derived cues implies that species-specific differences in the brain microenvironment can shape divergent microglial phenotypes. For example, variation in local signals between rodents and humans may contribute to differences in baseline gene expression observed between species [95]. Nevertheless, transplanted human microglia can survive and exhibit surveillance behavior in murine brains, suggesting that the core environmental signals required for microglial support are conserved across species [96].

Notably, in the context of severe CNS injuries or neuroinflammation, infiltrating peripheral monocytes can partially adopt microglia-like properties [22]. However, these peripherally derived cells remain transcriptionally distinct from bona fide microglia and fail to recapitulate their functional roles [97]. Other external stimuli are gut microbiota-derived signals that shape microglial morphology, number, metabolism, and function via short-chain fatty acids such as acetate [98, 99].

This highlights the dual importance of both the developmental origin and the environmental niche in preserving microglial identity.

### Microglial regeneration after depletion or injury

Microglia exhibit remarkable regenerative capabilities following experimental depletion or CNS injury. In vertebrates, pharmacological ablation of microglia via CSF1R inhibitors triggers rapid repopulation from residual microglia. In mice, microglial numbers are restored to baseline levels within one to two weeks, driven by local proliferation of surviving cells rather than recruitment from peripheral progenitors [86, 92, 100, 101]. Fate-mapping experiments confirmed that newly generated microglia exclusively originate from preexisting CNS-resident cells, underscoring their intrinsic regenerative capacity. Comparable regenerative responses are observed in zebrafish: following CSF1R inhibition, microglial numbers rebound to normal levels within two weeks, mirroring the regenerative dynamics observed in mammals [102].

Injury-induced microglial regeneration also follows a conserved pattern across vertebrates. Focal damage to the CNS induces local microglial proliferation, recruitment, and reconstitution of the affected tissue. In mice, microglia proliferate robustly for approximately two days following nerve injury [89]. Notably, clonal tracking reveals a shift from random, stochastic self-renewal during homeostasis to targeted clonal expansion during injury repair, reflecting an adaptive proliferative response tuned to local damage [89]. In zebrafish, microglia respond within hours of injury by migrating to the lesion site, clearing debris, and contributing to neurodegenerative processes [93, 103, 104]. Microglia also undergo local proliferation, and there is no major contribution of macrophages from the periphery [105]. While temporal kinetics and scale vary between species, the core ability of microglia to regenerate and restore tissue integrity is evolutionarily conserved.

## Molecular signature

### Core gene signature

Microglia are defined by a unique core gene expression signature that consistently marks homeostatic microglia in both the developing and adult CNSs [106, 107]. Transcriptomic studies have identified core genes in microglia. These include genes such as *P2ry12*, *Tmem119*, *Cx3cr1*, *Fcrls*, *Siglech*, *Sall1*, and *Hexb*, which are minimally expressed by peripheral macrophages or other brain cells [14, 72, 108]. These genes encode purinergic receptors, chemokine receptors, immunoregulatory membrane proteins, and transcriptional regulators involved in sensing, surveillance, and maintenance of microglial identity.

For example, P2RY12, a purinergic receptor, mediates microglial motility and responsiveness to ATP and ADP signals [109]. It is robustly expressed in homeostatic microglia across vertebrates but is downregulated upon activation [14, 16, 109]. TMEM119, a transmembrane protein recently implicated in amyloid-beta clearance [110], is specifically expressed in parenchymal microglia and has become a widely used marker in both human and mouse tissues [15]. SALL1, a zinc-finger transcription factor, is essential for preserving the microglial phenotype; in mice, its deletion [111] or reduced expression following disruption of a key regulatory enhancer [112] causes a transcriptional shift toward an inflammatory macrophage-like state. The lysosomal microglial molecule HEXB is delivered to neurons to ensure the presence of the neuronal membrane-derived ganglioside GM2 [113]. The absence of HEXB in both mice and humans causes a fatal neurodegenerative disorder called Sandhoff disease [114].

**Fig. 2:**
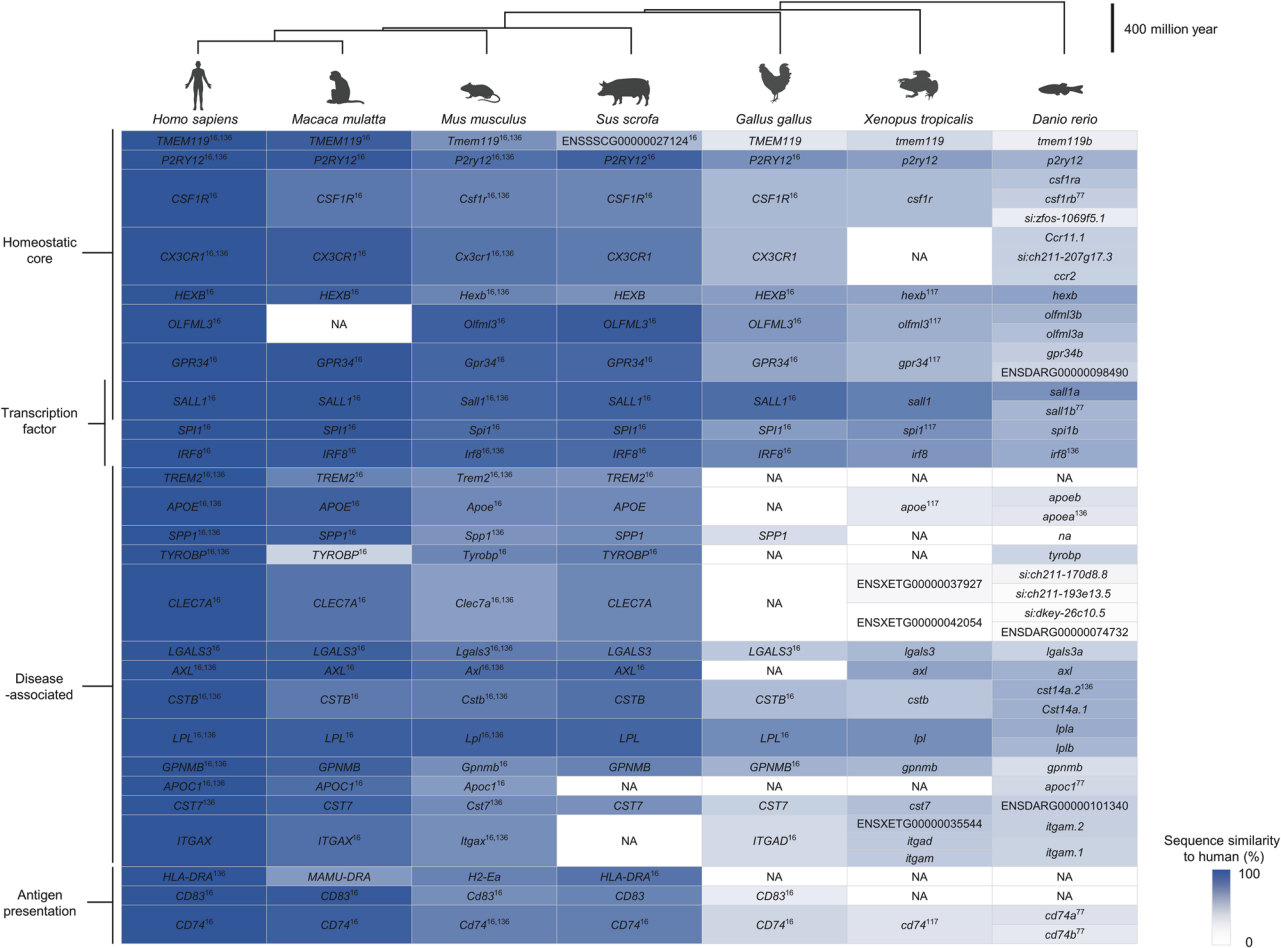
Molecular signatures of microglia across species. The heatmap displays orthologous genes for each animal species, with color intensity indicating their sequence similarity to the corresponding human gene. Orthologous gene and sequence similarity data were obtained from the Ensembl Genome Browser (https://www.ensembl.org/index.html). The phylogenetic tree of the animals reviewed in this study was generated via phyloT (https://phylot.biobyte.de/). NA, no ortholog available

Comparative studies revealed that these and other core genes are evolutionarily conserved from fish to mammals, although their sequence similarities vary (Fig. 2). Developing zebrafish microglia display transcriptional profiles closely resembling those of mammalian microglia, including orthologs of key mammalian signature genes [115]. For example, larval zebrafish microglia express purinergic receptors, Toll-like receptors, complement receptors, and MHC class II genes in patterns analogous to those of mouse microglia [105]. These observations suggest that fundamental microglial functions—such as purinergic sensing, phagocytosis, and antigen presentation—are supported by a conserved genetic toolkit that emerged early in vertebrate evolution.

A cross-species single-cell RNA-sequencing study encompassing more than ten vertebrate species, from zebrafish to nonhuman primates, defined a conserved microglial core gene program that includes orthologous genes involved in glia–neuron interactions and surveillance signaling [16]. Despite over 450 million years of evolutionary divergence, this conserved expression profile underscores the ancient and stable identity of microglia as CNS-resident macrophages. Nevertheless, species-specific differences in transcription do emerge atop this core program. While microglial gene expression in humans broadly overlaps with that in rodents, interspecies differences exist—particularly in genes related to disease susceptibility and immune signaling [77]. For example, primate microglia show higher baseline expression of genes in the complement cascade and lipid metabolism pathways than rodent microglia do [16]. Specifically, human microglia express higher levels of immune receptors such as *TLR*, *Fcγ*, and *SIGLEC* than mouse microglia do [116], highlighting human-specific immune and regulatory adaptations. Moreover, age-associated transcriptional changes show limited overlap between species [116], indicating that human and mouse microglia follow distinct molecular aging trajectories. Notably, microglial heterogeneity also varies by species: in most mammals studied, microglia harbor a relatively uniform population under homeostatic conditions, whereas human microglia display greater transcriptional diversity even in healthy individuals [16, 91]. This broader range of expression states may reflect the increased complexity of human CNS demands.

Despite such variation, foundational identity genes—including *P2RY12*, *TMEM119*, *HEXB*, and *SALL1*—are expressed in microglia across multiple species, including zebrafish, frogs, chickens, pigs, mice, monkeys, and humans [16, 117–119]. Notably, these core markers are expressed across the brain and developmental stages in both mice and humans, although their expression levels may vary in response to the regional environment [37, 106, 120–123]. Thus, the core gene signature of microglia is a robust and evolutionarily conserved molecular network, defining microglia across vertebrates despite species-specific modulation.

### Disease-associated gene signature

While microglia in healthy conditions share a conserved homeostatic signature, disease or injury induces transcriptomic reprogramming—resulting in reactive gene expression states that display both parallel and species-specific distinctions [124]. In mammalian models of neurodegenerative disease, microglia frequently transition into a phenotype known as “disease-associated microglia (DAM)” [125–127]. This state was first described in an Alzheimer’s disease (AD) 5xFAD mouse model, where a subset of microglia near amyloid plaques upregulated genes involved in lipid metabolism, phagocytosis, and antigen presentation (e.g., *Apoe*, *Trem2*, *Axl*, *Lpl*, *Ctsd*, and *Cd74*) while concurrently downregulating homeostatic markers such as *P2ry12* and *Tmem119* [128, 129]. Similar neurodegenerative microglial signatures have been identified in other models of disease, including Parkinson’s disease (PD), multiple sclerosis (MS), and amyotrophic lateral sclerosis (ALS) [130–132], suggesting that these reactive states represent a conserved program triggered by neuronal damage.

Importantly, human studies revealed overlapping features. In the brains of AD patients, microglia proximal to pathology exhibit elevated expression of genes such as *APOE*, *SPP1*, *APOC1*, and *HLA-DR*, alongside a reduction in core homeostatic genes—indicative of a phenotypic shift toward activated states [133–137]. Temporal tracking of microglial activation at single-cell resolution reveals distinct, stage-specific phenotypes during neurodegeneration [138]. Single-cell RNA sequencing analyses of samples from patients with other neurodegenerative disorders, such as PD, MS, and ALS, have similarly identified activated microglial clusters marked by genes such as *APOE*, *GPNMB*, *SPP1, LPL*, and *HLA-DR*, many of which overlap with mouse DAM signatures, albeit with differing proportions and kinetics [91, 139, 140].

Although vertebrate models for chronic neurodegeneration are limited to mammals, cerebroventricular microinjection of amyloid-β42 in zebrafish has been used to induce Alzheimer-like pathology [141]. In this model, single-cell RNA sequencing of microglia revealed the upregulation of genes such as *apoeb*, *apoc1*, and *ctsd*, with gene enrichment analysis highlighting pathways conserved with human AD microglia, including MHC protein binding, lysosomal processes, iron homeostasis, and energy metabolism [142]. Thus, the core features of disease-associated microglial status—the gain of genes involved in phagocytosis, lipid handling, and antigen presentation—appears to be broadly conserved across vertebrate species.

Despite this conservation, cross-species comparisons revealed significant differences in baseline and reactive microglial gene expression profiles. For example, the expression of neurodegenerative disease susceptibility genes in humans is more strongly correlated with that in macaques than with that in rodents, reflecting closer evolutionary relatedness [16]. Furthermore, primate microglia intrinsically express higher levels of risk genes for neurodegenerative diseases, such as *APOE*, *SORL1*, and *APOC1*, than mouse microglia do [77].

The transcriptional responses of microglia during neurodegeneration differ markedly across species. In mammalian models, microglia frequently upregulate proinflammatory and oxidative pathways that can exacerbate chronic CNS pathology [143]. However, recent xenotransplantation studies revealed that human microglia exhibit distinct activation profiles when exposed to AD pathology. Specifically, human microglia engrafted into 5xFAD mouse brains show disease-associated profiles similar to those of mice but display more pronounced responses to amyloid-β plaques, including the upregulation of HLA antigen presentation pathways [144]. This highlights species-specific response programs even in the same pathological context.

In contrast, zebrafish microglia exposed to neurodegenerative stimuli do not prominently express inflammatory mediators. Instead, they upregulate genes associated with proliferation [105], which is consistent with the remarkable regenerative capacity of the zebrafish CNS [93, 145]. These divergent responses underscore how evolutionary pressures may have shaped microglial programs to match species-specific physiological needs—favoring rapid tissue repair in regenerative species and modulating immune responses in long-lived organisms.

In conclusion, while microglia across species possess a conserved immunological repertoire for detecting and responding to CNS insults, the disease-associated transcriptional programs they deploy are species-tuned. Recognizing and dissecting these differences is essential for improving the translatability of preclinical models and for designing microglia-targeted therapies that are effective in human neurodegenerative conditions.

## Morphology

### Homeostatic state

In the absence of pathology, microglia across all vertebrate species adopt a highly ramified morphology characterized by branched processes radiating from a small central soma [146, 147]. This archetypal structure enables continuous environmental surveillance and represents a conserved hallmark of homeostatic microglia. However, cross-species comparisons reveal significant variability in the complexity of microglial arbors [16]. Quantitative morphological analyses have shown that marmoset microglia exhibit the most elaborate branching among mammals, whereas human microglia display intermediate complexity, challenging the assumption that microglial morphology scales linearly with brain size or evolutionary advancement [16]. This contrasts with other glial types, such as astrocytes, whose size and arborization increase with brain complexity across species ranging from rodents to primates [148]. Factors such as local gene expression programs, regional circuit architecture, and the CNS microenvironment all contribute to shaping microglial morphology [149]. These influences suggest that structural complexity is not simply a function of evolutionary advancement or brain size. Instead, the absence of a consistent phylogenetic gradient across species implies that microglial branching patterns are fine-tuned to meet the specific physiological demands and neural landscapes of each organism.

Despite these interspecies differences in morphological complexity, the core behavioral features of homeostatic microglia are remarkably conserved. In all vertebrates studied, microglia maintain a stable ramified form while continuously surveying their territory through dynamic extension and retraction of processes [146]. This behavior has been visualized in vivo across species ranging from zebrafish larvae [150] and frogs [151] to rodents [152] and even in human microglia xenografted into mouse brains, which retain their characteristic surveillance dynamics [96]. These findings underscore that the motile, sentry-like behavior of resting microglia is an evolutionarily conserved trait essential for maintaining CNS homeostasis.

### Activated state

Upon activation, microglia undergo pronounced morphological remodeling characterized by the retraction and thickening of processes and the adoption of an ameboid shape with an enlarged cell body [146]. This transformation, marked by a swollen soma and reduced, stubby branches, is a well-recognized hallmark of microglial responses to CNS injury, infection, or other immunological stimuli.

Although this qualitative shift from a ramified to ameboid morphology is broadly conserved across vertebrate species, the degree, kinetics, and recovery trajectory of activation can differ markedly. Comparative studies suggest that factors such as brain size, age, and environmental context modulate the morphological dynamics of microglial activation [153]. For example, following viral infection, both mice and marmosets display microglial activation with ameboid morphology; however, the extent of process retraction and the duration of activation vary [154]. In mice, microglia tend to clear the infection and return to the ramified state more quickly. In contrast, marmoset microglia exhibited a more prolonged activation phase with distinct morphological features, potentially reflecting the complexity and scale of the primate brain. Human‒mouse chimeric models have provided further insight into cross-species responses. In a study using mice engrafted with human induced pluripotent stem cell (iPSC)-derived microglia, systemic lipopolysaccharide administration resulted in significant reductions in process length, arbor complexity, and volume in both human and mouse microglia, demonstrating conserved activation capacity in a shared CNS milieu [155].

These findings collectively illustrate that while the fundamental morphological response to activation is conserved, the magnitude and temporal dynamics of microglial remodeling vary among species. Larger brains may pose unique logistical and metabolic challenges to microglial activation, and species-specific environmental adaptations likely shape the reactivity and resolution profiles of these resident immune cells.

## Functions

### Roles in physiology

#### Immune surveillance and homeostasis

A fundamental role of microglia across vertebrate species is the continuous surveillance of the CNS microenvironment. In mammals, studies have demonstrated that ramified microglia constantly extend and retract their processes to monitor the surrounding parenchyma for signs of disturbance [6, 156, 157]. In mice, two-photon microscopy revealed that these dynamic movements occur even under homeostatic conditions [152, 158].

Importantly, this behavior is not exclusive to mammals. In zebrafish larvae, microglia similarly exhibit active patrolling behaviors, moving their processes and migrating within neural tissue [150, 159]. This conserved surveillance activity reflects the shared role of microglia as sentinels that maintain CNS homeostasis across vertebrate species.

Microglia are equipped with a conserved molecular toolkit that enables this surveillance. Both zebrafish and mammalian microglia express pattern recognition receptors and purinergic receptors such as P2RY12, which mediate ATP-induced process motility [105, 109]. These receptors allow microglia to detect “danger” signals, cellular distress, or injury-associated molecular patterns, indicating that the core surveillance mechanisms are evolutionarily ancient.

Notably, the intensity and pattern of microglial surveillance vary across developmental stages or species. In mice, process motility is robust during early development but gradually diminishes with age [160]. Similarly, zebrafish microglia show high surveillance activity during the larval stage, but process dynamics and migratory behaviors decrease as the CNS matures [161]. This age-related decline may be correlated with the downregulation of genes associated with microglial motility and migration during development [115].

Taken together, these findings highlight that the role of microglia as dynamic surveyors of the neural microenvironment is highly conserved throughout vertebrate evolution. Their constant vigilance enables rapid detection of homeostatic perturbations, including apoptotic cell death, synaptic remodeling, or microbial invasion, and initiates timely responses to preserve CNS integrity.

#### Phagocytic clearance of debris

Microglia serve as professional phagocytes and are tasked with the essential function of clearing cellular debris [23]. They actively engulf and digest apoptotic cells, dead neurons, and other unwanted material to maintain tissue integrity and neural health [6]. This phagocytic role is particularly crucial during development, when widespread programmed cell death occurs throughout the CNS. Microglia are the primary cells responsible for the removal of these apoptotic elements. This developmental clearance function has been well documented across vertebrates, including zebrafish [161], frog [151], chicken [162], pig [163], mouse [164], rat and macaque [165], and human [35]. These findings highlight the conserved role of microglia in sculpting the developing nervous system by removing dying cells.

Despite this conservation, species differences in the capacity and efficiency of microglial phagocytosis have been reported. For example, in zebrafish, microglia exhibit strikingly efficient phagocytic behavior. Nearly all apoptotic cells in the developing zebrafish brain are found within microglia, suggesting rapid and near-complete clearance of cellular debris [150, 161]. In contrast, the developing mouse brain often contains apoptotic remnants that have not yet been engulfed, indicating relatively slower or more spatially constrained clearance processes [166].

This difference may reflect differences in brain size, cellular turnover, and spatial organization rather than a fundamental variation in microglial capacity. In mice, for example, the greater volume of neural tissue and episodic waves of neuronal apoptosis may overwhelm local phagocytic capacity, leading to transient accumulation of debris [23]. Thus, while the phagocytic function of microglia is evolutionarily conserved, their efficiency and dynamics are shaped by species-specific neurodevelopmental contexts and anatomical constraints.

#### Synaptic pruning and circuit remolding

In addition to their classical role in clearing dead cells, microglia are recognized as critical regulators of synaptic pruning and broader circuit remodeling during development [167]. In the mammalian CNS, a surge of synapse elimination occurs during early postnatal life to refine emerging neural networks, and microglia play an active role in mediating this process [168, 169]. Seminal studies in mice have shown that microglia engulf synaptic structures tagged by complement proteins, thereby eliminating weak or excess synapses [170, 171]. Pioneering studies in mice have demonstrated that microglia engulf synaptic elements tagged by complement proteins, facilitating the removal of weak or superfluous synapses [170, 171]. For example, genetic ablation of the complement receptor CR3 or its upstream activator C3 results in persistent excess synapses and impaired eye-specific segregation in the mouse visual system, underscoring the functional relevance of microglia-mediated pruning in circuit refinement [19, 170]. However, there is still debate about the specificity of microglia for these phenotypes, since mice deficient in microglia resulting from the deletion of the fms-intronic regulatory element (FIRE) in the *Csf1r* locus exhibit normal synapse numbers and synaptic maturation [172, 173].

This synapse-sculpting function of microglia may extend beyond rodents. Early histological studies in cats reported a microglial association with remodeling of the corpus callosum and visual tracts during development, suggesting conserved roles in shaping interhemispheric connectivity [174]. More recently, zebrafish studies have offered mechanistic insights into how microglia contribute to CNS development beyond synaptic pruning. In vivo imaging of zebrafish larvae revealed that microglia dynamically patrol developing neural structures and make selective contact with nascent myelin sheaths as early as 3 days post-fertilization (dpf) [175]. Genetic ablation of microglia via *csf1ra*/*csf1rb* double mutants led to abnormal myelin morphologies by 10 dpf [176], reminiscent of the phenotypes observed in CR3-deficient mice [19].

Importantly, zebrafish microglia preferentially target longer or malformed sheaths for lysosomal degradation, suggesting a selective quality control mechanism [176]. This targeted engulfment may rely on evolutionarily conserved “eat-me” signals [177]. For example, exposure of phosphatidylserine (PS) on abnormal myelin fragments acts as a clearance cue, engaging multiple PS receptors, including BAI1, TIM1, and AXL [178, 179]. Disruption of these receptors in combination substantially reduces myelin engulfment by microglia in zebrafish, mirroring the complement-mediated synaptic tagging observed in mammals [176].

Complement-dependent axonal pruning has also been demonstrated in amphibians. In developing frogs, two-photon live imaging revealed that microglia actively engulf axonal segments during early neural circuit formation [151]. Experimental enhancement of the complement C3 pathway significantly increased axon pruning at the level of individual axons, whereas overexpression of the complement inhibitor CD47, a membrane-bound “do not eat me”, reduced microglial engulfment and suppressed axonal pruning [151].

Microglia also contribute to circuit refinement in a constructive manner by facilitating synaptogenesis. In adult mice, microglia promote learning-associated synapse formation, partly via the release of brain-derived neurotrophic factor (BDNF). Inducible deletion of BDNF in microglia via the *Bdnf*^fl/fl^; *Cx3cr1*^CreER^ system impaired motor learning and reduced dendritic spine formation, highlighting a critical role for microglia-derived BDNF in activity-dependent synaptic plasticity [20]. Nevertheless, there is debate regarding whether microglia genuinely synthesize and release BDNF in vivo, as some recent studies have failed to detect *Bdnf* expression in microglia under physiological or pathological conditions [15, 180, 181]. In developing mice, microglial contacts have been shown to induce dendritic filopodium formation and stabilize nascent spines, directly influencing neuronal connectivity [182].

Although direct experimental evidence for synaptic modulation by microglia outside mammals remains limited, the presence of conserved molecular pathways—including purinergic and complement signaling—in zebrafish, birds, and mammals [16] suggests that microglial participation in shaping neural circuits is a widespread vertebrate feature. Together, these findings support a dual role for microglia in sculpting the CNS: they eliminate excessive or aberrant synaptic and structural elements while also promoting the growth and stabilization of appropriate connections. This balance of pruning and support is likely essential for achieving functional brain architecture across species.

#### Myelinogenesis and myelin maintenance

In addition to refining synaptic connectivity, microglia also contribute to the structural maturation of axonal insulation. In addition to synapses, they actively participate in the remodeling of myelin sheaths, a process that likewise depends on precise elimination and maintenance mechanisms to ensure optimal circuit performance.

In developing zebrafish, microglia-deficient csf1r mutants exhibit supernumerary and malformed myelin sheaths that fail to be removed efficiently, leading to disorganized myelin patterns and reduced conduction efficiency [176]. These findings demonstrate that microglia are required to sculpt nascent myelin during early development, ensuring correct sheath number and morphology.

In mammals, microglia are dispensable for initial myelin ensheathment but are essential for the later phases of myelin growth and maintenance [183]. Selective depletion of microglia in mice causes hypermyelination, myelin outfolding, and progressive demyelination with age, accompanied by metabolic alterations in oligodendrocytes and reduced myelin turnover. Similar myelin abnormalities are observed in human white matter carrying biallelic CSF1R mutations [65, 183, 184], supporting an evolutionarily conserved role for microglia in sustaining myelin integrity across species.

Together, these studies reveal that microglia act not only as sculptors of developing myelin but also as long-term maintainers of its structural and metabolic stability. The convergence of zebrafish, mouse, and human findings underscores a conserved principle by which microglia preserve efficient axonal insulation and CNS function.

#### Support of neurogenesis and developmental progression

Microglia contribute to neurodevelopment beyond synaptic pruning and play vital roles in neurogenesis, axonal growth, and vascular development. During the embryonic and early postnatal periods in mammals, microglia colonize the brain early enough to regulate neuronal progenitor cell proliferation and differentiation [185]. In the developing mouse brain, microglia phagocytose neural progenitor cells in regions such as the cerebral cortex and retina, effectively trimming the progenitor pool to optimize neuronal output [165, 186]. In parallel, microglia also support neuronal survival through trophic signaling. For example, in the developing mouse cortex, layer 5 pyramidal neurons require microglia-derived insulin-like growth factor 1 (IGF1) for survival; depletion of microglia leads to increased neuronal apoptosis [187]. Recent work using human brain organoids further supports this trophic function, showing that microglia-derived IGF1 promotes the proliferation of progenitors in the developing forebrain region that generates GABAergic neurons [188]. Together with findings from the mouse cortex, these findings extend the role of microglial IGF1 from supporting cortical neuron survival to promoting interneuron production in the developing human brain. These dual actions—eliminating excess cells while supporting appropriate cells—highlight the fine-tuning function that microglia play during CNS development.

Microglial regulation of neurogenesis persists into adulthood. In mammals, neurogenic niches such as the hippocampus and subventricular zone continue to produce new neurons throughout life [189]. In the adult mouse hippocampus, microglia selectively phagocytose apoptotic newborn neurons, a process shown to be critical for the survival and maturation of functional neurons [190]. This clearance prevents unnecessary inflammation and helps maintain an optimal environment for neuronal integration, reinforcing the importance of microglial homeostatic surveillance beyond early development.

In addition to mammals, the relationship between microglia and neurogenesis appears to be evolutionarily conserved but exhibits species-specific adaptations. Organisms with high regenerative capacity, such as zebrafish, display microglial behaviors that actively support neuronal regeneration. Zebrafish are capable of lifelong neurogenesis and robust CNS regeneration, far surpassing mammals in this respect [191]. In this context, pharmacological depletion of microglia impaired the regeneration of retinal neurons, underscoring their essential role in facilitating neurogenic recovery in fish [192]. These findings collectively illustrate that microglia are deeply integrated into the cellular networks guiding brain development, neurogenesis, and tissue plasticity across vertebrate species.

## Roles in pathologies

### Response to CNS trauma

In vertebrates, microglia are among the earliest responders to CNS trauma and rapidly migrate to the site of injury in response to purinergic and chemokine cues [152]. Upon arrival, microglia initiate debris clearance, synapse stripping, and cytoprotection, aiming to minimize excitotoxic damage and preserve surrounding neural circuits [193]. These acute functions are conserved across species, underscoring the evolutionarily ingrained role of microglia as immediate responders to neural insult.

However, striking interspecies differences emerge in the aftermath of this acute phase. In mammals, the microglial response frequently transitions into a chronic inflammatory state that may impede repair and exacerbate long-term neuropathology. In rodent and human models of traumatic brain injury, microglial activation persists for weeks to months after injury, with activated cells accumulating around lesions [194–196]. Even three months post-injury, the levels of inflammatory mediators such as IL-1β and tumor necrosis factor (TNF) remain elevated in murine brains [197]. Notably, early-phase pharmacological depletion of microglia does not ameliorate structural damage in the injured brain [198]. In contrast, microglial depletion during the chronic phase attenuates trauma-associated pathology and improves histological and behavioral outcomes [199, 200]. These findings suggest that while early microglial activity may be beneficial, prolonged activation contributes to secondary injury cascades.

In contrast, zebrafish display markedly different trajectories. Microglial activation following injury is transient and efficiently transitions into a regenerative program. Suppressing the immune response via dexamethasone impairs both neurogenesis and regenerative repair in CNS tissue [145], indicating that a properly timed inflammatory response is essential for recovery. Moreover, zebrafish deficient in microglia, such as those lacking the transcription factor Irf8 [62], exhibit increased lesion volume and impaired regenerative capacity after traumatic brain injury [201]. These data highlight the essential, pro-regenerative roles that microglia play in injury recovery in highly regenerative species.

Taken together, these cross-species comparisons suggest that evolution has shaped microglial responses to trauma to meet species-specific needs. In long-lived mammals, prolonged inflammation may serve as a defense against latent infections or ensure neural stability, but at the cost of reduced regenerative potential. In contrast, regenerative species such as zebrafish prioritize rapid resolution of inflammation to enable tissue regeneration, providing a distinct model of microglial adaptability in CNS repair.

### Contribution to neurodegenerative diseases

Microglia play pivotal roles in neurodegenerative diseases such as AD [202–204]. Through their ability to sense, clear, and respond to pathological proteins, they can exert both neuroprotective and neurotoxic influences depending on the disease stage and context [203, 205, 206].

In the early stages of AD, microglial phagocytosis is thought to contribute to the clearance of aggregated amyloid-β, thereby slowing disease progression [206, 207], although this early protective role remains debated, possibly owing to variations among experimental models or designs [208, 209]. Multiple lines of evidence support this neuroprotective role. Single-cell RNA sequencing combined with in situ hybridization revealed that microglia with strong phagocytic signatures preferentially localize near Aβ plaques in both mouse and human brains [128]. Similarly, fate mapping integrated with single-cell profiling demonstrated spatial and functional dichotomies between plaque-associated and nonplaque microglia [129]. In mouse hippocampal slice cultures, depletion of microglia or inhibition of amyloid-β42 phagocytosis increased neuronal death [210], underscoring the importance of efficient clearance mechanisms.

A critical receptor mediating this process is TREM2, which directly recognizes soluble amyloid-β oligomers [211, 212]. In AD patients, TREM2 variants with reduced amyloid binding affinity are associated with increased AD risk [211, 213–215], and human stem-cell-derived microglia-like cells lacking TREM2 show markedly decreased amyloid β uptake [216]. Consistently, deficiency or loss-of-function mutations in TREM2 lead to increased plaque burden and neuronal loss in mouse models [217, 218], confirming that microglia-mediated clearance is essential for early neuroprotection. Human–mouse chimeric models have further clarified this cross-species function: deletion of TREM2 in human microglia transplanted into AD-like chimeric mice severely impaired phagocytosis [219].

Microglia also protect neurons by forming a physical barrier around plaques. These processes wrap tightly around amyloid-β deposits, limiting the spread of neurotoxic amyloid-β42 fibrils and shielding adjacent axons [220]. This barrier function appears to be conserved in humans, as carriers of loss-of-function mutations in TREM2 display disrupted microglial encapsulation of plaques accompanied by severe axonal dystrophy [221].

Despite these early protective effects, aberrant or prolonged microglial activation can exacerbate neuronal damage in later disease stages [203]. Pharmacological depletion of microglia or blockade of microglial proliferation in late-stage AD mouse models reduces neuroinflammation, prevents neuronal loss, and ameliorates behavioral deficits [222, 223].

Several mechanisms underlie this transition. One involves complement-mediated synapse loss: in AD mouse models, the complement component C1q—produced primarily by microglia [224]—tags synapses, marking them for elimination. The activation of downstream complement components such as C3 subsequently drives excessive synaptic pruning by microglia, resulting in cognitive decline [225]. Complement receptor 1 (CR1) is one of the major AD risk factors associated with faster cognitive decline [226, 227]. The CR1 variants provide additional binding sites for C3b/C4b [228], and human iPSC-derived microglia carrying these variants exhibit increased phagocytic activity toward synaptoneurosomes [229].

In addition to aberrant synaptic engulfment, microglia contribute to neuronal injury through the release of inflammatory mediators [230]. For example, active caspase-1 expression is elevated in the brains of AD patients, and its deletion in mice with mutations associated with familiar AD results in enhanced amyloid-β clearance and is largely protected from the loss of spatial memory [231]. Similarly, type I interferon signaling is upregulated in the brains of AD patients, and blocking this pathway—either by antibody treatment or microglia-specific deletion of the interferon receptor IFNAR1—protects against synaptic loss in 5xFAD mice [232, 233].

Human imaging studies further support this biphasic model of microglial function. PET imaging with translocator protein (TSPO) and other tracers reveals early and late peaks of microglial activation during the AD trajectory [234], followed by a decline as the amyloid-β burden plateaus [235]. Elevated microglial activation is detected even at prodromal or preclinical stages, with higher TSPO binding in individuals who show slower cognitive decline, which is consistent with an early protective phase [236]. Conversely, microglial activation increases again after the transition from mild cognitive impairment to Alzheimer’s disease [237], correlating with cognitive deterioration and predicting clinical progression, which is consistent with the detrimental phase [238–240].

Although relatively few studies are available, zebrafish models provide complementary evidence for conserved microglial responses in neurodegeneration. Injection of amyloid-β42 into the zebrafish brain triggers microglial activation and IL-4 production, which promotes neural progenitor proliferation and neurogenesis, potentially reflecting their enhanced regenerative capacity [141]. Notably, shorter Aβ fragments fail to elicit microglial activation [141], indicating a specific response to toxic amyloid species [241, 242]. In contrast, amyloid-β42 exposure enhances cytokine signaling in microglia, including the IL-1 and TNF signaling pathways [142], reminiscent of the inflammatory profiles observed during later disease stages in mammalian AD.

Together, these observations delineate a dual-phase role of microglia in AD—protective during early amyloid deposition through phagocytosis and barrier formation but progressively detrimental as chronic activation promotes complement-mediated synapse loss and neuroinflammation. The balance between these phases likely differs across species owing to variation in microglial gene expression, inflammatory thresholds, and regenerative potential. Understanding these dynamics across experimental models and humans is essential for developing strategies that preserve beneficial microglial functions while mitigating their late-stage neurotoxicity.

## Conclusions

Cross-species analyses of microglia highlight an evolutionarily conserved yet functionally diverse population of CNS-resident immune cells. From their embryonic origin and transcriptional regulation to their roles in surveillance, synaptic pruning, and neurodegeneration, microglia have emerged as central determinants of brain health and disease across vertebrates. While core identity features—such as yolk sac origin, self-renewal capacity, and homeostatic gene expression—are widely conserved, significant species-specific differences emerge in morphology, gene expression programs, and responses to injury or pathology. These distinctions reflect adaptation to varying brain architectures, life spans, and regenerative potentials.

**Fig. 3:**
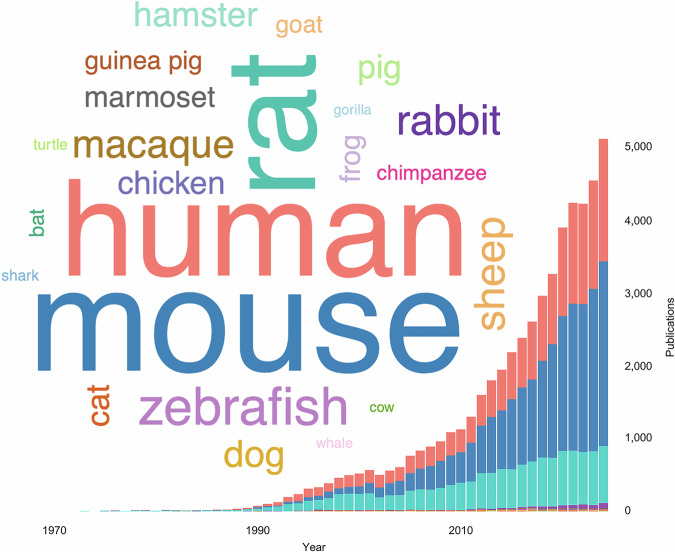
Trends in the species used in microglial research. The word cloud shows the cumulative PubMed publication frequency by species, and the stacked bar chart shows the annual number of publications per species. The color corresponds to the species across both visualizations

Microglial research itself has expanded dramatically over recent decades. A growing diversity of species—ranging from zebrafish to macaques—has been incorporated into microglial studies, as evidenced by recent trends in publication frequency (Fig. 3). While rodent models remain dominant, the increasing inclusion of nontraditional and nonmammalian species reflects both the limitations of conventional models and the recognition that key aspects of microglial biology may be more effectively contextualized in an evolutionary framework. Recent studies further illustrate these limitations, revealing resident macrophage populations that share microglial identities but reside in tissues such as the peripheral nervous system (PNS), fetal skin, and testis [39]. Notably, a distinct microglia-like population in the PNS appears to exist only in humans and other large vertebrates but is absent from rodents [40]. Together, these findings underscore the importance of comparative approaches to understand the full spectrum of immune lineages with microglial molecular features and ontogeny through evolution.

Despite this growing appreciation, key questions remain. One major challenge lies in interpreting microglial function under nonhomeostatic conditions, where cross-species differences in immune tone, neurogenic capacity, and aging trajectories become increasingly pronounced. In experimental models of disease, microglial responses can differ substantially even among closely related species, raising concerns about the translatability of rodent findings to humans. This issue is further compounded by the heterogeneity of disease models: whereas human neurodegenerative conditions develop over decades through complex gene‒environment interactions, most animal models rely on acute, artificial insults or genetic modifications. These differences limit our ability to draw definitive conclusions about the contributions of microglia to pathogenesis.

To address these challenges, future comparative studies can benefit from strategic designs that minimize confounding variables. Ideally, using parallel models under well-controlled conditions allows direct comparison of microglial responses to equivalent stimuli. For example, exposing mice, zebrafish, and primates to standardized inflammatory or neurodegenerative triggers—while applying shared methodologies such as single-cell RNA sequencing or live imaging—may help disentangle species-specific responses from technical artifacts. Harmonizing experimental protocols across laboratories, including animal housing, tissue processing, and analytical workflows, can further reduce variability and improve the interpretability of cross-species datasets.

Another emerging solution lies in the use of naturally occurring disease models. Certain animals—such as aged nonhuman primates, dogs, or even cats—spontaneously develop pathologies resembling human neurodegenerative disease [243, 244]. These systems avoid the artificial acceleration or overexpression often observed in transgenic models and may more closely recapitulate the timing and trajectory of human disease. Similarly, interspecies chimeras offer a promising approach for probing human-specific microglial features within a controlled, experimental environment.

The environmental context is another critical variable that shapes microglial phenotype and function. Standard laboratory conditions—especially in rodents—often employ specific-pathogen-free (SPF) housing, which profoundly alters the host immune system. “Wildling” mice, which are rederived into naturalistic environments, present more mature immune profiles that more closely resemble those of adult humans [245, 246]. The incorporation of such models into cross-species analyses may enhance translational relevance and better capture the spectrum of microglial states found in natural settings.

In conclusion, cross-species comparisons of microglia offer powerful insights into both shared and divergent strategies of CNS immune regulation. This study reveals not only the ancient evolutionary origins of microglia but also their remarkable plasticity in adapting to species-specific demands. The integration of findings from rodents, zebrafish, nonhuman primates, and human-derived systems will be essential for developing microglia-targeted therapies that are robust, scalable, and relevant to human health. By acknowledging and leveraging interspecies differences, we may ultimately translate basic discoveries into clinically meaningful interventions for neurodevelopmental and neurodegenerative disorders.
